# Color Comparisons and Interpersonal Variation

**DOI:** 10.1007/s13164-016-0323-2

**Published:** 2016-11-25

**Authors:** Nat Hansen

**Affiliations:** 1 Department of Philosophy, University of Reading, HumSS Building Whiteknights, RG6 6AA Reading, UK; 2 Stanford University Humanities Center, Stanford, CA USA

## Abstract

An important challenge to color objectivists, who hold that statements concerning color are made true or false by objective (non-subject-involving) facts, is the argument from interpersonal variation in where normal observers locate the unique hues. Recently, an attractive objectivist response to the argument has been proposed that draws on the semantics of gradable adjectives and which does not require defending the idea that there is a single correct location for each of the unique hues (Gómez-Torrente (2016) *Noûs* 50(1): 3–40),. In (Hansen (2015)), I argued that the recent objectivist response doesn’t apply to comparative occurrences of color adjectives, so a revised, comparative, version of the argument from interpersonal variation remains a powerful objection to certain types of objectivism. In this paper, I address several unsatisfactory objectivist replies to the comparative version of the argument from interpersonal variation, and offer what I think is a more plausible objectivist reply to the comparative argument from interpersonal variation.

## Introduction: Interpersonal Variation and Color Metaphysics

Experimental participants with “normal” color perception who are shown monochromatic lights or chips that reflect lights of different dominant wavelengths disagree to a surprising extent when asked to identify which lights or chips are examples of “unique” hues.^1^ The unique hues (unique red, blue, green, yellow) were identified by the German psychologist Ewald Hering (1920) as those hues that are phenomenologically “pure” or “unmixed”: unique red appears to contain no blue or yellow, unique yellow appears to contain no blue or green, unique blue appears to contain no red or yellow, and unique green appears to contain no blue or yellow. The most dramatic variation occurs with judgments about unique green: different experimental participants identify stimuli ranging anywhere from 490nm to 555nm as unique green. This is such a broad range that it overlaps with the area within which some participants locate unique blue (458nm–495nm) on one side and unique yellow (544nm–594nm) on the other (Kuehni 2004, p. 161).^2^

Block (1999, p. 43), discussing the variation in judgments about unique green among 50 normal perceivers reported in Hurvich et al. (1968), draws the following table that represents the variation:

**ᅟ:** ᅟ

5 subjects located unique green at	490 nm
11 at	500 nm
15 at	503 nm
12 at	507 nm
5 at	513 nm
2 at	517 nm

Block describes the “upshot” of the variation as follows: “if we take a chip that any one subject in this experiment takes as being unique green, *most* of the others will see it as at least slightly bluish or yellowish” (p. 43).^3^

This widespread disagreement about where to locate the unique hues has played an important role in arguments that target theories of color that identify colors with physical properties of objects (surface spectral reflectances, for example). Cohen (2009, Ch. 2) sets out a “master argument form” for arguments from perceptual variation, of which the argument from interpersonal variation in the location of the unique hues is one version. What follows is one way of partially filling in a version of Cohen’s master argument form (Cohen^2009^, p. 24) with facts regarding variation about the location of the unique hues (green in particular):

1. *Variation*: Normal observers disagree in their judgments about where to locate unique green when presented with colored stimuli of a particular wavelength. An observer, Wy, may judge that a 500nm stimulus is unique green, for example, while another observer, Zed, may judge that the same stimulus is not unique green (because it is too bluish).
2. *Ecumenism*: There is no good reason to think that one normal observer’s judgment that *x is unique green* (not at all bluish or yellowish) is veridical while another normal observer’s judgment that *x is not unique green* (because it is bluish) is not. Either both judgments are veridical, or neither is.^4^
3. *Anti-irrealism*: The idea that neither judgment is veridical (perhaps because colors aren’t properties of external objects) is implausible.
4. So (from 1-3) we have reason to think that both judgments (*x is unique green* and *x is not unique green*) are veridical.
5. But if both judgments are veridical, then they must not contradict one another.
6. The best to way to reconcile the apparently contradictory judments (Wy: *x is unique green* vs. Zed: *x is not unique green*) is…

There are competing ways of completing the final part of the argument from interpersonal variation and avoiding the appearance of problematic contradictory judgments about the location of the unique hues. For example, relationalists (Cohen 2009e.g.) complete the argument by arguing that colors are relations between objects and various parameters (including perceiving subjects), and since the parameters of the two seemingly contradictory judgments are different, the contradiction is merely apparent.^5^ And color “pluralists” or “selectionists” (Allen 2009; Kalderon 2007; Mizrahi 2006) complete the argument from interpersonal variation by arguing that different observers pick up on (‘select’) different, non-relative, properties possessed by the stimulus. The veridicality of the seemingly contradictory judgments is due to different observers “perceiving different families of colors” (Kalderon 2007, p. 593) that inhere in the stimulus.^6^

## Gómez-Torrente’s Semantic Response to the Argument from Interpersonal Variation

Gómez-Torrente (2016) rejects both relationalism and selectionism as satisfactory ways of preserving the veridicality of apparently contradictory judgments about the location of the unique hues, and he rejects a standard objectivist response that involves denying premise (2), ecumenism. He rejects Kalderon’s version of selectionism on the grounds that it “doesn’t appeal to any sort of relativization that could make the properties [*green but neither somewhat yellow nor somewhat blue* and *green but somewhat blue*] compatible” (p. 7), and so can’t offer a satisfying way of explaining how apparently contradictory judgments about the location of the unique hues can both be veridical. I think Kalderon’s notion of colors belonging to “different families” might be capable of addressing this worry, but I won’t explore that option here.^7^

Gómez-Torrente rejects relationalist theories that hold that colors are constituted by a relation to an experiencing subject because there is, he thinks, “an extended… impression that the color properties are physical properties ‘out there’, not involving subjects or perceptual relations at all” (p. 6).^8^ And he thinks that subjectivist relationalism implies that color properties have unintuitive modal properties—specifically, that it predicts that statements like (1) should sound true, because on the subjectivist relationalist view, (1) expresses something like (2) (which is true), when in fact it should sound false (according to Gómez-Torrente’s intuitions and those of his informants):

1. If my eyes were just a bit different, this stimulus would stop being unique green.
2. If my eyes were just a bit different, this stimulus would stop being unique green (to me).^9^

Gómez-Torrente also rejects attempts to respond to the argument from interpersonal variation that deny premise (2), *ecumenism*. It is possible to deny premise (2) by holding both that (a) we don’t have any independent and well-motivated reason for singling out some particular judgment about unique green as veridical at the expense of all other conflicting judgments and that (b) there is (still) such a correct judgment. It’s possible to hold both (a) and (b) if it’s the case that we just don’t *know* which judgment is veridical and which isn’t (Byrne and Hilbert 2003, p. 17; Tye (2006)). Gómez-Torrente(2016, p. 7) finds this reply “very unconvincing”, because:

We have no clue as to what fact of the matter, if this is to be describable in terms of the relevant known physics and physiology, could make BG [the subject who judges that a particular stimulus is not unique green] or UG [the subject who judges that the same stimulus *is* unique green] or some other subject be in the right to the exclusion of the others. Moreover, in part because of this, but also insofar as purely linguistic judgments are concerned, neither BG nor UG would seem to have made any mistake in their use of color words. It is hard to avoid the conclusion that objectivist accounts that respected the impression that all the subjects are equally right would enjoy a definite advantage over this kind of reaction.

Gómez-Torrente sets out to give an objectivist account that has such an advantage by respecting *ecumenism*, and which avoids the problems he identifies with both subjectivist relationalism and Kalderon’s pluralism. His account offers a linguistic response to the argument from interpersonal variation, by showing how it is possible for there to be judgments that disagree about the location of the unique hues, and which look contradictory, but which can simultaneously be veridical.^10^ He does so by drawing an analogy between color adjectives (“green”, “blue”, etc.) and gradable adjectives that project objects onto scales that order objective magnitudes.^11^

**Fig. 1:**
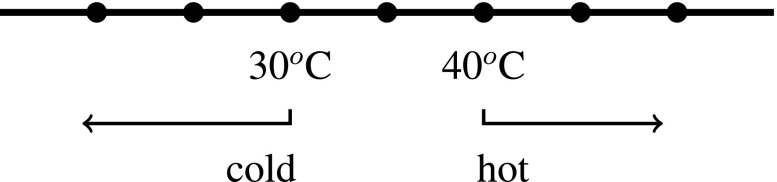
Wy’s standards on the dimension of *temperature*

**Fig. 2:**
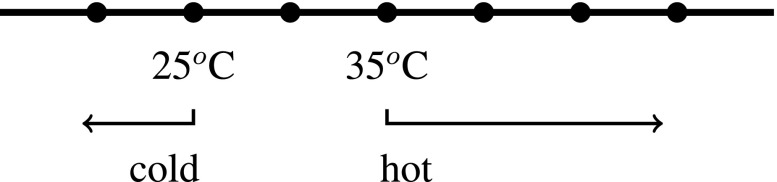
Zed’s standards on the dimension of *temperature*

A standard semantics for gradable adjectives like “tall” and “hot” associates such expressions with functions from objects to degrees on a scale (Bartsch and Vennemann 1972; Cresswell 1977; Kennedy 1999). A scale is a set of degrees ordered along some dimension (height, temperature, etc.). Relative adjectives like “tall” and “hot” require a value for a contextually variable *standard* in order to generate a traditional property of objects (a function from objects to truth-values): When Wy and Zed describe something as “hot”, their intentions fix different standards that something must meet or exceed on the scale of hotness in order to count as hot. 35 degree water may count as hot according to Zed’s standard, but may not count as hot according to Wy’s standard, for example.^12^ Figures 1 and 2 show that by setting the standards for what counts as “hot” in different places, Wy and Zed can disagree over whether something is hot (e.g., water that is 35 ^*o*^C): Wy will judge that it isn’t hot, and Zed will judge that it is.^13^

The adjectives “tall” and “hot”, though they have standards that may differ between subjects, still order objects along scales that measure objective dimensions: namely *height* and *temperature*, respectively. When standards are set in different places on the scale of hotness (which is a way of ordering the underlying objective dimension of temperature), it is possible for two people to make apparently contradictory judgments (*x is hot* and *x is not hot*), that are nevertheless both veridical. When Zed says “x is hot” (in a context where his standard for what counts as hot is being at least 35 ^*o*^C), he says something true just in case x has a temperature of at least 35 ^*o*^C. When Wy says “x is not hot” (in a context where his standard for what counts as hot is being at least 40 ^*o*^C), what he says is true just in case x doesn’t have a temperature of at least 40 ^*o*^C. So when x is 35 ^*o*^C, Wy can truly say “x is not hot” and Zed can truly say that “x is hot”.^14^ Even though the way content is fixed involves subjective aspects of speakers (their intentions, e.g.), the content that is thereby fixed can be objective (“x is hot” is true just in case x has a temperature of at least 40 ^*o*^C, e.g.) (Gómez-Torrente 2016, pp. 8–9).

**Fig. 3:**
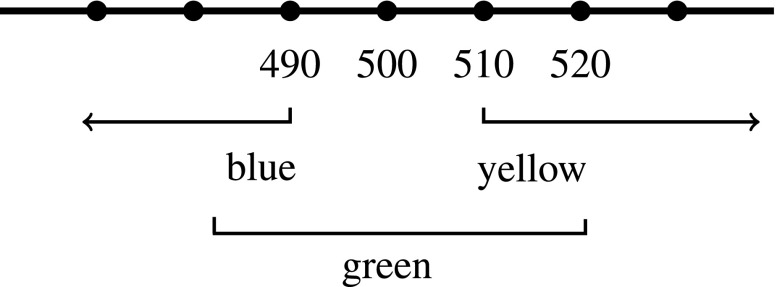
Wy’s standards

**Fig. 4:**
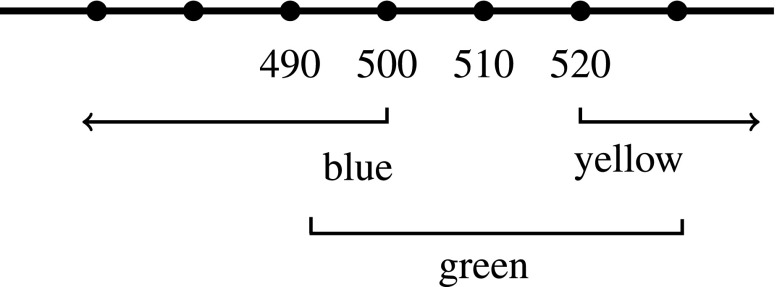
Zed’s standards

Gómez-Torrente’s central claim is that color adjectives operate the same way as “hot” and “tall”: the appearance of interpersonal disagreements over whether something is unique green or not stem from locating the relevant standard in different places on a scale of hue that is a way of ordering degrees on an underlying objective dimension.^15^ Different degrees on the objective dimension can then be ordered into a scale of hues, as in Figs. 3 and 4 (which are based on Gómez-Torrente (2016 figure 2)).

If color adjectives like “green” have varying standards in the same way that “tall” and “hot” do, then disagreements over whether some hue is unique green or not can be explained as disagreements over where the standards of greenness are located on the scale of hues. So, for example, Wy may judge that a stimulus, x, consisting of 500nm monochromatic light *is unique green*, while Zed judges that *x is not unique green* (because it’s too blue). In so judging, Wy would be saying something true just in case x is located between 490nm (his standard for what makes something count as minimally blue) and 510nm (his standard for what makes something count as minimally yellow) (see Fig. 3). Zed’s judgment that *x is not unique green* would be true just in case x is not located between 500nm (his standard for what makes something count as minimally blue) and 520nm (his standard for what makes something count as minimally yellow). (See Fig. 4).

If that approach is right, then the phenomenon of intersubjective variation in judgments about unique hues is compatible with a (non-selectionist) objectivist view of color—it can be understood as the result of locating the relevant standards for, e.g., “blue”, “green”, and “yellow” in different places on the scale of hues.

## A Comparative Version of the Argument from Interpersonal Variation

Gómez-Torrente provides a way for an objectivist about color to preserve *ecumenism* in the argument from interpersonal variation, while preserving the idea that the reference of particular uses of “unique green” (when combined with a contextually variable standard) is a non-subject-relative property—having a particular dominant wavelength, for example. While I find Gómez-Torrente’s argument appealing, in Hansen (2015) I argued that there is a variant of the argument from interpersonal variation that makes it difficult for the (non-selectionist) objectivist to embrace both the idea that uses of color adjectives refer to purely objective properties and that the conflicting judgments of normal observers are veridical. The variant of the argument that I gave involves *comparative* judgments of hue (*x is greener than y*), rather than categorical judgments (*x is unique green*).

If Wy thinks that h is unique green, and Zed doesn’t, then they will also disagree about whether h is *greener* than certain other stimuli or not. For example, Wy will find a 500nm stimulus greener than a 510nm stimulus, and Zed will find a 510nm stimulus greener than a 500nm stimulus (see Figs. 3 and 4). This is not just a difference in where Wy and Zed locate their standards for greenness on a single, shared hue scale—it is a difference in how Wy and Zed *order* hues on *different* scales of greenness. This marks an important difference between the scales associated with color adjectives and the scales associated with adjectives like “tall” and “hot”.

To bring out the difference, consider the fact that if Wy and Zed disagree about which of two objects is *taller* or *hotter*, then one of them has to be making a mistake. That’s because there’s no room for subjectively different orderings of the degrees associated with the objective dimensions of height and temperature. Imagine how odd “tall” and “hot” would look if it were possible for two people to (veridically) disagree about the ordering of degrees on the dimensions of height and temperature:

- Wy: 40 ^*o*^C is hotter than 35 ^*o*^C.
- Zed: 40 ^*o*^C is not hotter than 35 ^*o*^C.

Only if Zed is using “hotter” (or one of the other words in the sentence) to mean something idiosyncratic could what he says be veridical.

That’s not the case with color adjectives. Wy and Zed can disagree about the proper *ordering* of objects in terms of how green they are while both still making veridical judgments. That means that they are operating with different scales of greenness (since a scale is a particular ordering of a set of degrees along a dimension).^16^ So the analogy that Gómez-Torrente draws between color adjectives and adjectives like “tall” and “hot” breaks down in an important way.

Proponents of the subject-relativity of color might point out that color adjectives look closer to predicates of personal taste (which measure objects along subject-relative dimensions if anything does) in that both involve subjective variability in scales, not just standards on scales. There is nothing problematic about two subjects who rank the “tastiness” of foods in different ways, for example. But it would be hasty to conclude from the fact that color adjectives involve subjective variability in scales that those scales don’t order a single underlying objective dimension (of dominant wavelengths, for example) in different ways. Consider a type of adjective that serves Gómez-Torrente’s purposes better than “tall” and “hot”, namely adjectives that (a) involve an objective underlying dimension, and (b) involve a focal point standard that objects can diverge from *in two directions*. “Mild” is such an adjective: it involves a focal point that objects can diverge from in two directions on the dimension of temperature: x can be milder than y because it is colder than y (if y is hot), or because it is hotter than y (if y is cold).

On the standard semantics for gradable adjectives that Gómez-Torrente invokes, a comparative construction (“x is hotter than y”, “x is greener than y”) is true just in case x has a greater degree on the relevant scale (*hotness*, *greenness*) than y. How might the semantics of comparative occurrences of adjectives like “x is milder than y” be handled? One straightforward possibility would be to give the truth conditions of such statements in terms of “closeness” to the relevant standard: Wy’s judgment that *500nm is greener than 510nm* is true just in case 500nm is closer to what he considers to be unique green (500nm) than 510nm is. Assuming that identity is maximal closeness, then because 500nm is what Wy judges to be unique green, his judgment *500nm is greener than 510nm* is true. Zed’s judgment that *510nm is greener than 500nm* is true just in case 510nm is closer to what he considers to be unique green (510nm) than 500nm is. Since a 510nm stimulus is what Zed judges to be unique green, then his judgment is true. And it seems that the truth conditional content of both judgments can be spelled out purely objectively, in terms of the objective properties 500nm and 510nm, with subjective features of Wy and Zed only playing a role in fixing the relevant contents.

But (I argued in Hansen (2015)), a key aspect of the truth conditional content of the comparative color judgments, the notion of *closeness* to unique green, can’t plausibly be defined without invoking a subject-relative property, even once interpersonal differences in what counts as unique green are fixed to objective properties (500nm or 510nm, e.g.). That’s because there can be interpersonal variation in judgments about which stimuli are *closer* to unique green.

**Fig. 5:**
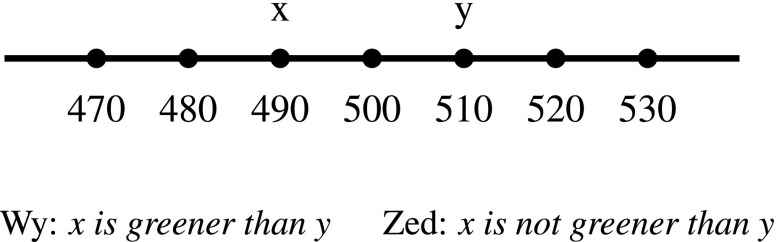
Disagreement in comparative judgments (*across unique green*)

For example, assume that Wy and Zed are participating in an experiment where they are presented with monochromatic light stimuli, and that both judge that light at 500nm is unique green.^17^ But even if they locate unique green in the same place on the objective dimension of wavelength (500nm, say) they can disagree about whether, e.g., light at 490nm (shifted towards blue) or at 510nm (shifted towards yellow) is *greener* (closer to unique green). (See Fig. 5).

If both Wy and Zed’s judgments in such a situation are veridical, then that means that *closeness* to unique green can’t be defined in terms of distance along the dimension of wavelength. A partial explanation for the fact that this type of comparative variation is possible is the existence of lower-level physiological differences between subjects (screening pigments or numbers of cone types, e.g. (Malkoc et al. 2005, p. 2155)), or higher-level differences between subjects (past visual experiences, e.g. (Hinks et al. 2007, p. 3371)), which would contribute to an explanation for why one subject might be more sensitive to differences on the blue side of unique green, while another might be more sensitive to differences on the yellow side.

So, assuming the veridicality of both Wy and Zed’s comparative judgments of hue, the truth conditions for comparative judgments of hue (*x is greener than y*) involve an element that looks like it can’t be spelled out in non-subject-involving terms, namely *closeness* to unique green. If that’s right, that would block Gómez-Torrente’s attempt to show that subjective features only play a content-fixing role and don’t contribute to the truth conditional content of judgments involving color adjectives. Whether the relevant notion of closeness can be spelled out in non-subject-involving terms will be discussed in detail in Section 5.

The traditional argument from interpersonal variation can therefore be reformulated in comparative terms, as follows:

1. *Variation*: Normal observers disagree in their judgments about where to locate unique green when presented with colored stimuli of a particular wavelength.
2. *Categorical variation implies comparative variation*: If normal observers disagree in their judgments about where to locate unique green, then normal observers disagree in their comparative judgments (*x is greener than y*) of greenness.
3. *Comparative variation*: Normal observers disagree in their comparative judgments (*x is greener than y*) of greenness.
4. *Comparative ecumenism*: There is no good reason to think that one normal observer’s judgment that *x is greener than y* is veridical while another normal observer’s judgment that *x is not greener than y* is not. Either both judgments are veridical, or neither is.
5. *Anti-irrealism*: The idea that neither comparative judgment is veridical (perhaps because colors don’t exist) is implausible.
6. So we have reason to think that both judgments (*x is greener than y* and *x is not greener than y*) are veridical.
7. But if both judgments are veridical, then they must not contradict one another.
8. The best to way to reconcile the apparently contradictory judments (*x is greener than y* vs. *x is not greener than y*) is…

If my argument about the ineliminability of a subject-relative element in defining “closeness” in the truth conditions of comparative color judgments were right, then Gómez-Torrente wouldn’t have a non-subjective way of completing the argument from interpersonal variation.^18^

## Objections and Replies to the Comparative Argument from Interpersonal Variation

Gómez-Torrente (2015) offers several responses to my comparative version of the argument from interpersonal variation. He summarizes his objections as follows:

The cases of disagreement on which [Hansen] builds his new subjectivist argument are, if they are not merely hypothetical, probably few and not evidently relevant as test cases for a semantic theory; in particular, they strongly suggest the possibility that some semantic indeterminacy is lurking. Finally, and most importantly, even if there is no semantic indeterminacy involved, the disagreements in question are not usable by the subjectivist, for they are relevantly different in kind from the disagreements about positive [non-comparative] color statements that subjectivists have often employed of late.

In this section, I’ll discuss each of Gómez-Torrente’s three objections:

- the (merely) hypothetical nature of comparative disagreement
- lurking semantic indeterminacy
- comparative disagreements are different in kind from disagreements about positive [non-comparative] color statements

Concerning (a), the hypothetical status of the kinds of comparative disagreement that my argument depends on, Gómez-Torrente says “I am not aware of experimental evidence showing that there are actual disagreements of this very specific kind among normal perceivers, nor does [Hansen] provide references” (p. 3). That’s right. I wasn’t relying on any existing experimental studies that explicitly concern these types of disagreements (as opposed to disagreements about positive—non-comparative—judgments about the location of the unique hues). But there is evidence that I think makes it reasonable to predict that such disagreements exist. In his discussion of the experimental evidence of interpersonal variation in the location of the unique hues, Kuehni(2004, pp. 161–162) writes:

Among the conclusions [to be drawn from facts about interpersonal variation in the location of the unique hues] is that it is not justified, certainly not in case of green and red, to assume that a mean UH [unique hue] can be considered representative of humans. This raises questions about the degree of validity of color appearance models. While no explicit data have yet been published the impression is that for individual observers UH are not rotated one way or the other against the mean in a simple manner. It means that the perceptual distances between unique hues may vary to a smaller or larger extent by observer. This raises significant questions about color space scaling and color difference evaluation… In a given quadrant one observer may be significantly more or less sensitive to hue differences than another.

So even if observers agree about the location of unique green, we should expect that at least some will disagree about the perceptual differences in different quadrants of color space, which would lead directly to the kind of comparative disagreement that I discuss in my comparative version of the argument from interpersonal variation.

Gómez-Torrente also worries (b) that the kind of comparative disagreements that my argument relies on will be semantically indeterminate, and so not capable of providing a robust challenge to his theory. He argues, both for “mild” and for color adjectives, that comparative judgments give rise to indeterminacy when subjects are asked to make comparative judgments about stimuli that fall on either side of the relevant focal point standard. That is, there is no semantic fact of the matter when it comes to cross-focal point comparisons.

Consider cross-focal point comparative judgments about “mild”. Suppose that the living room is a mild 20 ^∘^C, while the kitchen is noticeably warmer (say 30 ^∘^C), and the bedroom is noticeably cooler (say 15 ^∘^C) than the living room. If asked to judge which is milder, the kitchen or the bedroom, Gómez-Torrente thinks that normal subjects should feel “hesitant and somewhat puzzled”, which he takes to be evidence that such comparisons are indeterminate. I agree that cross-focal point comparisons are (and feel) more cognitively complex than comparisons of stimuli that both fall on the same side of the focal point. It’s easy, for example, to judge whether an unbearably hot room or one that is slightly too hot is more mild, while it’s harder to judge whether a chilly room is more mild than one that is too hot. The cross-focal point judgment is harder to make because it involves working out not just a difference, but a difference of differences: which is greater, the difference between the hot room and the ideally mild room, or the difference between the chilly room and the ideally mild room? That’s a more cognitively complex task, which I think accounts for Gómez-Torrente’s felt difficulty about such judgments. But the cognitive complexity doesn’t make such judgments, whether about “mild”, or about color adjectives, indeterminate.

**Fig. 6:**
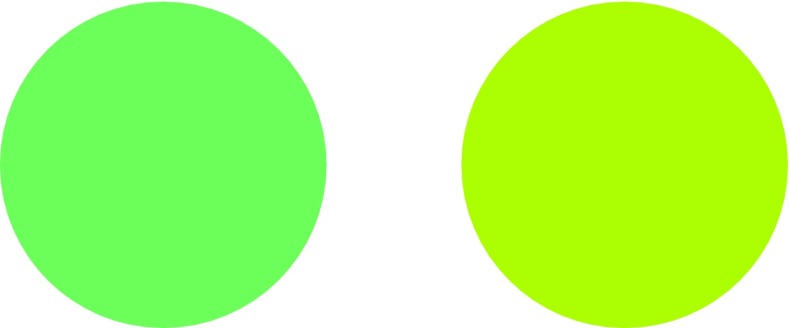
Cross-focal point comparative color judgment

I think an armchair experiment can help establish that cross-focal point comparative color judgments are not indeterminate, at least for some judges. Consider the pair of stimuli in Fig. 6, and ask yourself: which is greener?^19^

I think it’s not that difficult to judge which is greener (for me, the yellowish green circle is greener), which is evidence that cross-focal point judgments are not indeterminate in the way that Gómez-Torrente alleges.^20^

Finally, and “most importantly” (c), Gómez-Torrente thinks that there is a disanalogy between disagreements about comparative color statements and disagreements about positive statements. The purported disanalogy is that it is easier to see how, when two observers disagree in their comparative color judgments, one of them can be wrong. If that’s right, it would provide a way for Gómez-Torrente to deny *comparative ecumenism* while holding on to *ecumenism* in the non-comparative argument from interpersonal variation.

The disanalogy is that while it’s not plausible that certain normal perceivers are better at locating the unique hues, it *is* plausible that certain people can be better “at comparing distances between wavelengths”. This is just a case in which certain normal observers “can make hue discriminations at a higher level of acuity than others” (p. 5). For example, an especially acute observer who locates unique green at 500nm might be able to judge that a 509nm stimulus is greener (closer to unique green) than a 510nm stimulus, while a less acute observer (who also locates unique green at 500nm) would judge both stimuli to be equally green. It looks like there is a difference here that one observer is sensitive to that the other observer is simply missing.

I grant that some people can be better at comparing distances between wavelengths. After all, there’s an objective test to see how good they are at it. But in the case that I imagine (and which I think we have reason to think exists), the two observers making contradictory comparative judgments are disagreeing over which of two hues that are *objectively equidistant* from unique green is greener. In such a situation, the objective measure of whose judgment is “closer” to unique green is not going to help decide things—unless one is willing to maintain that *both* judges are simply wrong about their comparative judgments, due to the fact that both hues are equally green because they are objectively equidistant from what is judged to be unique green.^21^ But this idea is in tension with the fact, which Gómez-Torrente acknowledges, that there are asymmetries in judges’ sensitivity to blue and yellow stimuli: a 10nm difference on the yellow side of unique green will not typically be perceived to be as close to unique green as a 10nm difference on the blue side of unique green.

Gómez-Torrente observes that, even if there is full determinacy in cross-focal point comparisons, the truth conditions for such comparisons will be “somewhat more complicated”, in such a way that, if correct, would make it possible to say that in the case of comparative disagreement that is central to my argument, in which both observers agree about the location of unique green but disagree about which of two objectively equidistant hues is greener, one of the observers is judging truly and the other is judging falsely. That would justify a denial of *comparative ecumenism* in the comparative version of the argument from interpersonal variation, blocking the argument.

The complication involves holding that the notion of “closeness” to unique green that is needed to give the truth conditions for comparative cross-focal point judgments (*x is greener than y*) must involve more than just measuring the objective distances (in nanometers, e.g.) between x and unique green and y and unique green and determining which is straightforwardly closer. Because “the yellowish greens probably occupy a smaller zone of the objective dimension of hues than the bluish greens” (p. 6), and we’re more sensitive to small differences in the yellow portion of the visible spectrum, the semantic notion of “closeness” required by his theory needs to incorporate a scaling factor that would apply to objective distances on the blue side of green to bring them into alignment with objective distances on the yellow side of green. Once the scaling factor is in place, it would be possible, for example, to say that, for anyone who locates unique green at 500nm, a 490 (bluish green) stimulus would be greener than a 510nm (yellowish green) stimulus, even though both stimuli are objectively equidistant from unique green (500nm), because the scaling factor applied to distance from green on the blue-green side would result in a bluish green 490nm stimulus being “closer” to 500nm than a yellowish-green 510nm stimulus.^22^

I don’t think this way of responding to the central case in the comparative version of the argument from interpersonal variation is satisfying. The scaling factor “f” that Gómez-Torrente builds into the truth conditions of comparative occurrences of color adjectives makes Wy’s judgment *490nm is greener than 510nm* come out true, and Zed’s judgment *490nm is not greener than 510nm* come out false. But where would “f” come from, if not from an average of individual observers’ relative sensitivities to objective differences in the blue-green part of the spectrum vs. their sensitivities in the yellow-green part of the spectrum? There is just as little reason to think of the scaling factor “f” that results from such an average as *correct* as there is to think that average locations of the unique hues are correct. So there’s no reason to embrace the idea that the scaling factor “f” could play a role in determining which of two apparently contradictory comparative judgments is true in the way Gómez-Torrente needs it to in order to defend his objectivist position against the comparative version of the argument from interpersonal variation.

## A Better Response to the Comparative Argument from Interpersonal Variation: a Subjective Scaling Factor and Multidimensionality

While I don’t think Gómez-Torrente’s objections to the comparative argument from interpersonal variation succeed, there is a response to the argument that seems compatible with Gómez-Torrente’s overall approach, but which he does not consider. The alternative response involves introducing another form of subjectivity into the way the truth conditions of comparative color statements are fixed, while maintaining the idea that the underlying dimension along which objects are compared in terms of hue is an objective magnitude. As indicated by the passage from Kuehni quoted above, the relevant scaling factor—needed to accommodate subjective variation in sensitivity to different parts of the spectrum—will itself likely vary across different observers. According to this proposal, subjective features will play a role not just in fixing what the focal point of greenness is, but also what the relevant scaling factor is for what counts as “closeness” on the bluish-green vs. yellowish-green sides of unique green.

**Fig. 7:**
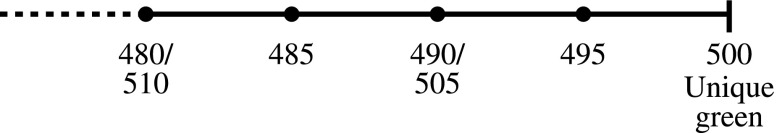
Subjective ordering of an objective dimension (*Zed*)

**Fig. 8:**
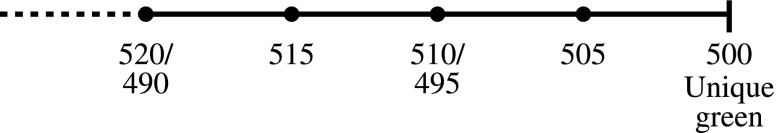
Subjective ordering of an objective dimension (*Wy*)

A comparative statement (*x is greener than y*) will be true, therefore, just in case x is closer to the relevant subjectively fixed unique green, where “closeness” to green itself a subjectively fixed property. Zed may judge that 490nm light is closer to unique green than 510nm light, because he is more sensitive to the yellow-green part of the spectrum than to the blue-green part of the spectrum, and Wy may judge the reverse because of the opposite sensitivity. But the resulting scales on which objects are compared involves purely objective magnitudes, as represented in Figs. 7 and 8.

Figure 7 is a scale that represents twice the sensitivity to the yellow-green part of the spectrum than to the blue-green part, and Fig. 8 represents the reverse. The two scales order the underlying dimension in subject-specific ways—other subjects will differ in terms of their relative sensitivities, and so will employ scales of greenness with different orderings of degrees—but the degrees on the scale remain objective magnitudes. Once such a scale is fixed, with 500nm as its maximum degree, the truth conditions of comparative statements are straightforward: *x is greener than y* is true just in case x has a greater degree on the relevant scale of greenness than y. According to the scale in Fig. 7, for example, 490nm light is greener than both 485nm light, and 510nm light. The important point is that not only is the maximum degree on the scale of greenness subjectively fixed, but the ordering of (objective) degrees on the scale is also subjectively fixed.

But why is it possible to generate subjectively variable orderings of hue if the underlying dimension of hue is just as objective as the underlying dimensions of height, temperature, and cost, for which it is not possible to generate such subjectively variable orderings? The answer is that hue, unlike height, temperature, and cost, is multidimensional.

In Hansen (2015), I considered a way of responding to the comparative version of the argument from interpersonal variation that invoked the idea of multidimensionality. My response went as follows:

There are adjectives, like “big”, that order objects along obviously objective dimensions, and for which there can be apparent disagreements about comparative judgments (of *bigness*), and for which the comparative judgments can both be veridical. Wy and Zed might, for example, disagree over who is bigger: Kareem Abdul-Jabbar (2.18m, 102kg), or Hulk Hogan (2.01m, 137kg). If Wy’s scale for “big” employs a scaling factor that assigns greater value to height than mass, then he can veridically judge that Abdul-Jabbar is bigger than Hulk Hogan. If Zed’s scale for “big” employs a scaling factor that assigns greater value to to mass than to height, then he can veridically judge the reverse. Apparent disagreement in comparative judgments about bigness can be explained in terms of different weightings assigned to the objective dimensions that produce the scale of bigness on which objects are ordered.

I dismissed this response, on the grounds that hue is a single dimension (as opposed to color in general, which is often modeled using three dimensions: hue, saturation, and lightness, for example), so I concluded that it wasn’t possible to hold that color adjectives (when they are only tracking hue) are multidimensional (Hansen 2015, p. 7). But, following Barceló (2016), I now think it is possible to think of hue judgments like “x is green” as involving two different objective dimensions: a green-rather-than-blue dimension and a green-rather-than-yellow dimension.^23^ Assuming that judgments about hue are a (subjectively variable) function of the degrees that objects have on both of those dimensions, then if two judges assign different weightings to those two dimensions in their comparative judgments, they can generate the subjective orderings of objective dimensions represented in Figs. 7–8, and thereby explain how they can disagree whether x is greener than y, even when they both agree about the location of unique green.^24^ That defuses the challenge posed by the comparative version of the argument from interpersonal variation.^25^

## Conclusion

In Hansen (2015) I argued that comparative hue judgments pose a problem for a promising objectivist response to the argument from interpersonal variation about the location of the unique hues. Gómez-Torrente (2015) offered three responses to my comparative argument. In this paper, I have given reasons to think those responses fail. But there is a better response to my previous argument, which explains the variation in comparative judgments of hue in terms of differing subjective weightings of objective underlying dimensions, thereby giving a more satisfying objectivist response to the comparative version of the argument from interpersonal variation about the location of the unique hues.

## Footnotes

1 See Hurvich et al. (1968) and Kuehni (2004) for the relevant experiments, and see Hardin (1988), Block (1999), and Cohen (2009) for discussion of the experiments’ philosophical significance.

2 Kuehni generates these large ranges from responses to different experiments. Allen (2010) worries that differences in experimental conditions across different studies, notably differences in light intensity, might problematize combining results in this way.

3 Note that Block refers to the observers in the Hurvich et al. (1968) experiment as responding to “chips”, but the experimental set up involved the observers viewing “the exit slit of a Farrand monochomator” through “a monocular short focus telescope”, and adjusting the wavelengths that the monochromator transmitted “until the test field he saw was a green that was neither yellowish nor bluish in appearance” (p. 67).

4 The “ecumenism” label comes from Cohen (2009, p. 24) via Logue (2016). Support for ecumenism comes from the failure to find any satisfying, non-arbitrary way of identifying which judgment about the location of unique green is veridical—for example in terms of the judgments of a “normal” or “standard” perceiver. See Cohen (2009, pp. 31–33) and Hardin (1988 ,pp. 67–82) for reasons in favor of thinking that there isn’t any such satisfying standard that identifies the correct location for the unique hues.

5 For example, two observers, Wy and Zed, who make apparently contradictory judgments about the location of the unique hues, turn out to be making judgments that are not in fact contradictory: *The 500nm stimulus is unique green according to standard W* vs. *The 500nm stimulus is not unique green according to standard Z*.

6
*The 500nm stimulus is unique green*_*family**W*_ vs. *The 500nm stimulus is not unique green*_*family**Z*_.

7 See Logue (2016, p. 216) for a discussion of this issue.

8 The intuition that experience presents colors as being non-relational is widely shared among philosophers of color. See Cohen (2009, pp. 154–159) for a list of those reporting the intuition, and for a compelling challenge to the significance of the experience of colors as non-relational for relational theories of color.

9 For what it’s worth, I don’t share the truth-value judgment reported by Gómez-Torrente and his informants about (1), but assessing the adequacy of Gómez-Torrente’s challenges to pluralism and subjectivist relationalism are not my main concern in this paper. Instead, I’m interested in Gómez-Torrente’s own promising semantic way of preserving the veridicality of both apparently contradictory judgments in the argument from interpersonal variation.

10 The remainder of this section and Section 3 are drawn from Hansen (2015), with some modifications.

11 Though his argument turns on features of color language, Gómez-Torrente argues that his conclusions about language extend to color experience on the “natural” supposition that “the color properties signified by color words are represented by color experience” (p. 4).

12 Gómez-Torrente draws on the degree-based semantics for gradable adjectives given in Kennedy (2007). For an application of that degree-based semantics to gradable color adjectives, see Kennedy and McNally (2010) and Hansen (2011).

13 Figures 1 and 2 are based on Gómez-Torrente (2016, figure 1).

14 The apparent disagreement between Wy and Zed in such a situation might be explained in terms of a disagreement over what the relevant standard for being hot is or should be, not what the temperature of the object is. See Plunkett and Sundell (2013) for a discussion of this kind of “metalinguistic” disagreement.

15 Gómez-Torrente wants to remain neutral between competing objectivist accounts of what the colors are, so he lets the variable *h* stand for a degree on whatever objective dimension (dominant wavelength, e.g.) is supposed to correspond to the hue dimension.

16 See Kennedy (2013, p. 267), who observes that “evaluative” predicates, such as predicates of personal taste, give rise to “faultless disagreement” with comparatives, while “dimensional” predicates (like “colder”/“richer”/“taller”/“heavier”/“older”) do not allow faultless disagreement in their comparative forms. See also Bylinina (2013), McNally and Stojanovic (2014), Solt (2016), and Umbach (2016).

17 See Volbrecht et al. (1997) for an example of an experiment evaluating the location of unique hues involving monochromatic light against different backgrounds.

18 Selectionism, however, would still provide an objectivist way of completing the comparative argument from interpersonal variation.

19 In the tikz rgb color system that I used to draw these circles, the blue-green circle has the values 0,1,0.6, and the yellow-green circle has the values 0.7,1,0. So in the rgb color system, they are almost equidistant (in the direction of blue and yellow, respectively) from unmixed green.

20 A referee reports that he or she was undecided about which circle is greener. That might be because the referee judges the circles to be *equally green*, which would explain the indecision in a way that is consistent with there being a determinate fact about cross-focal-point comparisons, or it might be because there is interpersonal variation in whether cross focal-point comparisons are determinate or not. See Hansen and Chemla (2016) for examples of interpersonal variation in the way gradable adjectives are applied.

21 Thanks to an anonymous referee for advocating this response.

22 This is my translation of the following passage in Gómez-Torrente (2015, p. 6): “On the other hand, in the imagined case of UG and UG ^*#*^, and utterance of *ν is greener than* *ξ* will be true just in case f ⋅ d _*g*_(h(*ν*)) < d _*g*_(h(*ξ*)), and so it will be true, given that f will be less than one. So UG’s utterance of *ν is not greener than ξ* will be declared false and UG ^*#*^’s utterance of *ν is greener than ξ* will be declared true”. In other words: There are two observers, UG and UG ^*#*^, who agree on the location of unique green (500nm, say), but who disagree whether *[490nm] is greener than [510nm]* (UG denies it and UG ^*#*^ affirms it). An utterance of *[490nm] is greener than [510nm]* will be true just in case the distance from unique green to 490nm, multiplied by a scaling factor less than one, is less than the distance from unique green to 510nm. Since the distance from 490nm to unique green (500nm), multiplied by a scaling factor less than one, *is* less than the distance from unique green to 510nm, an utterance of *[490nm] is greener than [510nm]* is true.

23 This possibility was also raised by Peter Pagin, Hanoch Ben-Yami, and Jérémy Zehr in discussion.

24 For a detailed semantic theory of multidimensionality that captures this kind of comparative variability, see Solt (2016). Solt herself observes that “the individual dimensions that underlie such [lexical] entries [for multidimensional adjectives] may themselves be objectively measurable; subjectivity derives from the potential for variation in the choice of these dimensions and how they are combined” (Section 5.3).

25 A referee wondered to what extent a relationalist, who holds that colors involve relations to perceiving subjects, would be threatened by this more complicated semantics for color comparisons. The proposed semantics for color comparisons doesn’t directly threaten a relationalist view of the colors, but it poses an indirect threat by offering an objectivist alternative that is consistent with the interpersonal variation data, which is standardly taken to support relationalism (bracketing alternative objectivist responses like selectionism). But the proposed semantic theory does not provide a response to other versions of Cohen’s “master argument form” for relationalism, where the variation is intra-subjective, or inter-species. So it does not constitute a comprehensive objectivist response to relationalism. See Gómez-Torrente (2016, p. 32 n. 2) for discussion.
